# Fecal Microbiome Alterations of Mice Following Immunization with Gold Nanoparticle Vaccines Against Enterohemorrhagic Escherichia coli

**DOI:** 10.21203/rs.3.rs-5146579/v1

**Published:** 2024-11-26

**Authors:** Sarah Bowser, Itziar Chapartegui-González, Alfredo G. Torres

**Affiliations:** The University of Texas Medical Branch; Karolinska Institutet; The University of Texas Medical Branch

**Keywords:** EHEC, Citrobacter rodentium, nanovaccines, microbiome, gold nanoparticles

## Abstract

**Background.:**

Enterohemorrhagic *Escherichia coli* (EHEC), a group of enteric pathogenic bacteria that is a major cause of human diarrheal disease, must interact with the diverse intestinal microbiome during colonization and subsequently overcome the environmental challenges to survive and cause disease. While this relationship, and how the microbiome modulates infection of EHEC, has been studied, it is less understood how the microbiome is impacted during treatment for an EHEC infection. One area that is notably lacking in knowledge is how vaccination can impact the intestinal microbiome composition, and therefore, influence vaccine efficacy. We previously developed vaccine formulations consisting of gold nanoparticles (AuNPs) conjugated to various EHEC antigens and tested them in small animal infection models using both EHEC and its murine counterpart *Citrobacter rodentium*. The goal of this study was to evaluate the relationship between these EHEC vaccines and their effects on the gut microbiome.

**Results.:**

We found that immunization with the vaccines or adjuvant-only control did not lead to major alterations in the composition of the fecal microbiome; however, there were measurable variations in individual mice within the same vaccine group housed in separate cages. Finally, immunization with one vaccine (AuNP-EscC) did prevent a decrease in the diversity of the fecal microbiome and an increase in detectable *C. rodentium* following infection compared to the control animals.

**Conclusions.:**

Overall, our small study argues in favor of evaluating the intestinal microbiome during vaccine development not just for EHEC, but for other enteric pathogens as well.

## BACKGROUND

The gut microbiota in mammals profoundly influences metabolic and nutritional processes, as well as the host's immune responses [1]. There is increasing evidence that links alterations in the microbiota with human diseases, from gastrointestinal to neurological pathologies. *Escherichia coli* is a commensal, non-pathogenic bacteria in human and other mammals’ intestinal microbiota; however, some pathogroups, such as Enterohemorrhagic *E. coli* (EHEC) can cause intestinal disease [2]. The interplay between EHEC, a human diarrheal illness-causing bacteria, and the intestinal microbiota during infection is an area of active investigation. It has been well reported that to coordinate the expression of its virulence factors, EHEC must interact directly with the intestinal environment and its residents, as well as recognize various microbiota-derived molecules, such as short chain fatty acids (SCFAs) [3, 4]. For example, butyrate, which is a SCFA produced in high concentrations in the large intestine primarily by *Firmicutes*, can increase expression of genes involved in EHEC adherence to intestinal epithelial cells [4, 5]. This attachment is mediated by enhanced expression of a specialized Type 3 Secretion System (T3SS), a virulence mechanism encoded by a pathogenicity island known as the Locus of Enterocyte Effacement (LEE) [2]. Additionally, EHEC is known to produce Shiga toxins (Stx) [2]. Stx acts by inhibiting protein synthesis and inducing apoptosis of endothelial cells [6]. These effects can be local in the intestines or in distant organs such as the kidneys if able to reach circulation. A high-fiber diet approach in mice was demonstrated to increase lethality and disease severity by EHEC following oral infection [7]. This diet not only increased intestinal butyrate and the expression of the Stx cellular binding target Gb3, which increased Stx-coordinated disease outcomes, but it also led to a reduction in commensal *E. coli*, providing a more favorable niche for EHEC colonization.

While the microbiome is known to promote pathogenesis of EHEC, there is also evidence that it is involved in resistance to infection. These mechanisms can be directly, either through nutrient competition or production of SCFAs or other molecules, or indirectly by modulating the intestinal immune response [4]. Conventional mice are typically resistant to EHEC infection and are only transiently colonized with minimal to no symptoms, despite evidence that the pathogen can adhere to mouse intestinal epithelial cells [8, 9]. This resistance is in part attributed to the composition of the microbiome [9]. Alteration of the mouse gut microbiome, either through antibiotic (streptomycin) treatment or with germ-free mouse strains, increases the susceptibility of mice to EHEC [9]. Additionally, the murine pathogen *Citrobacter rodentium*, which is an LEE-encoding bacteria that has been used extensively as a surrogate model of infection to study EHEC-related pathogenesis in mice, can disrupt the microbiome and cause dysbiosis to enhance its own colonization by mechanisms such as increasing oxygenation at the intestinal epithelial cell (IEC) interface [10]. These changes result in increased colonization of facultative anaerobes (like itself and other *Enterobacteriaceae*) and a reduction of commensal obligate anaerobes, further proving the role of commensal organisms in protection against invading pathogens [10].

Considering the ability of the microbiome to both promote and prevent EHEC colonization, which has also been demonstrated with other enteric pathogens [3, 4], prophylactic and post-exposure modulation of the intestinal micro-environment have been explored as potential therapies to limit infections. Examples of these methods include the administration of pre- and pro-biotics and fecal microbiome transplantation [11]. One area that has garnered less attention is the effects that vaccines have on the microbiome, and vice versa. Several studies, both in animals and humans, have indicated that the composition of the microbiome can impact vaccine immunogenicity, mostly due to its role in modulating immune responses [12]. Fewer investigations have examined vaccine-induced changes in the microbiome and whether these can influence efficacy. Studies in humans have largely focused on microbiome alterations following vaccinations against HIV-1 and SARS-CoV-2, while those in animals have included *Mycobacterium tuberculosis* and *Lawsonia interacellularis* [13]. Only one group has explored this area in the context of *E. coli* vaccinations, and they found that intranasally immunizing mice with three enterotoxigenic *E. coli* (ETEC) antigens, which are also conserved in some commensal bacteria, did not significantly alter the fecal microbiome composition [14].

Although EHEC poses a significant healthcare burden, being a major cause of diarrheal diseases in developed countries and South America, as well as being a leading cause of hemolytic-uremic children (HUS) in children due to Stx, there is currently no human vaccine available to protect against EHEC [15]. Previously, our lab developed novel vaccines using gold nanoparticles (AuNPs) conjugated to EHEC antigens and tested them in mice using both EHEC and *C. rodentium* (expressing Stx2d toxin) as infection models [16-19]. In these studies, we concluded that some vaccine formulations (AuNP-EscC) could successfully limit colonization of non-Stx2d-producing *C. rodentium* [19]. Therefore, the purpose of the current study was to determine if these AuNP vaccines induced changes to the murine intestinal microbiome and if AuNP-EscC could affect infection-induced microbiome alterations compared to non-immunized animals.

## METHODS

### Bacterial Strains and Growth Conditions.

*Citrobacter rodentium* strain used in this study (ATCC DBS771) was purchased from the American Type Culture Collection (ATCC). It was routinely grown in Luria-Bertani (LB) broth, supplemented with antibiotics for selection (chloramphenicol and kanamycin). For animal infections, a final concentration of 10^6^ CFU per dose was prepared in PBS, as previously described [19].

### Vaccine formulation

The cloning and purification of the EHEC antigens EscC and Eae (intimin gamma protein) were performed as described [19, 20]. The synthesis of AuNPs was performed using the Turkevich method [19, 21]. Gold-nanoparticles with the correct size and structure were conjugated with the proteins and finally resuspended in PBS for animal use [19].

### Animal Studies

Female 5-to-7-week-old C57BL/6 mice were purchased from Jackson Laboratories (Bar Harbor, ME, USA), housed in microisolator cages under pathogen-free conditions and maintained on a 12 h light cycle, with food and water available *ad libitum*. All animal protocols were reviewed and approved by the Institutional Animal Care and Use Committee (IACUC) of The University of Texas Medical Branch (Protocol #2112077). Mice were immunized subcutaneously (s.c.) three times at 2-week intervals with either AuNP-EscC, AuNP-Eae, or AuNP-EscC + AuNP-Eae (*n* = 6 per group), along with detoxified cholera toxin B subunit (Sigma, Cream Ridge, NJ, USA) and 2% Alhydrogel^®^ (InvivoGen, San Diego, CA, USA) as adjuvants [19]. Control mice (*n* = 6) were given unconjugated AuNPs with the same concentration of adjuvants. For vaccine efficacy assessment and microbiome evaluation following infection, *n* = 6 mice from AuNP-EscC or the control group were infected with 10^6^ CFU of *C. rodentium* DBS771 using a feeding method of infection, as previously described [19].

### Fecal Collection and Processing

Fecal samples were collected from mice in all groups before vaccine administration (pre) and 2 weeks following the last immunization dose (post). Feces were also collected from AuNP-EscC-immunized and control mice at 3- and 7-days post-infection (dpi). Feces were frozen at −80°C until processing. To extract microbial DNA, feces were processed using the QIAGEN QIAamp PowerFecal Pro DNA kit according to manufacturer instructions. Purified DNA samples were stored at −20°C until further use.

### Transcriptomic analysis

High-throughput sequencing of the bacterial 16S ribosomal RNA gene was performed using gDNA isolated from each sample. Sequencing libraries for each isolate were generated using universal 16S rRNA V3-V4 region primers [22] by Illumina 16S rRNA metagenomic sequencing library protocols. The samples were barcoded for multiplexing using Nextera XT Index Kit v2. Sequencing was performed on an Illumina MiSeq instrument using a MiSeq Reagent Kit v2 (500-cycles). To identify the presence of known bacteria, sequences were analyzed using the CLC Genomics Workbench 8.0.1 Microbial Genomics Module1. Reads containing nucleotides below the quality threshold of 0.05 (using the modified Richard Mott algorithm) and those with two or more unknown nucleotides or sequencing adapters were trimmed out. All reads were trimmed to 264 bases for subsequent operational taxonomic unit (OTU) classification. Reference-based OTU picking was performed using the SILVA SSU v119 database [23]. Sequences present in more than one copy but not clustered to the database were placed into de novo OTUs (97% similarity) and aligned against the reference database with 80% similarity threshold to assign the “closest” taxonomical name where possible. Chimeras were removed from the dataset if the absolute crossover cost was three using a k-mer size of six. Microbiome Analyst 2.0 (https://www.microbiomeanalyst.ca/) was used for visualization and to perform the statistical analysis of the microbiome data [24, 25]. Alpha diversity was measured using Shannon and Chao1 entropy (OTU level). Beta diversity was calculated using the Bray-Curtis diversity measure (OTU level). The significance was obtained by a permutation test.

## RESULTS

### AuNP-protein immunization and adjuvant-only treatment led to some differences in the composition of the fecal microbiome of mice, with only minimal changes in the overall diversity.

**Figure 1:**
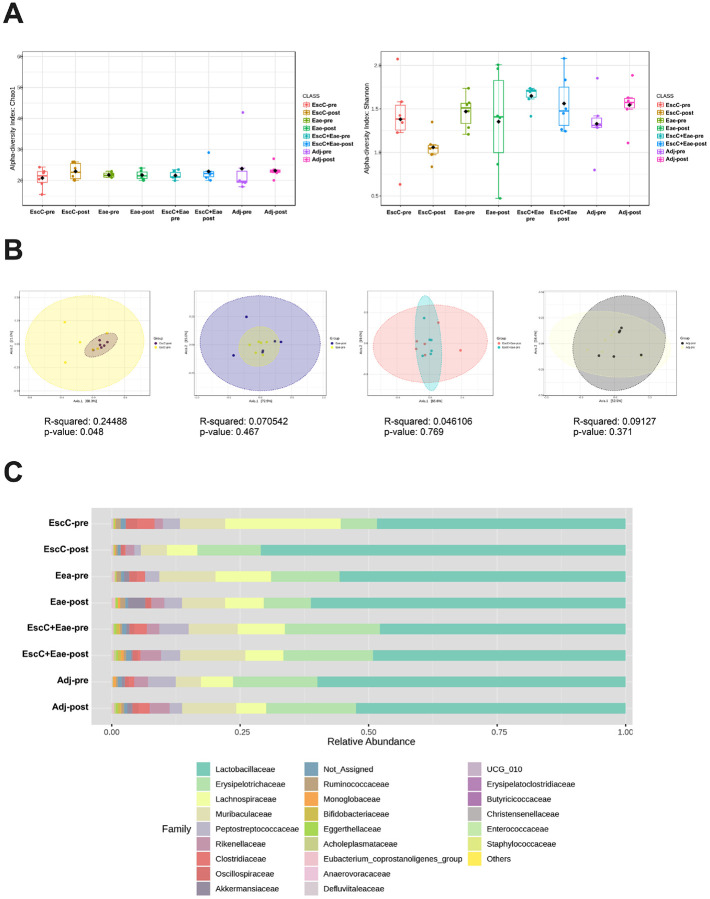
Alterations in mice fecal microbiome before and after immunization with either AuNP-proteins or AuNP-unconjugated. (**A**) Alpha-diversity of bacterial transcripts at the family level -pre and after -post immunization with AuNP-proteins or AuNP + adjuvant only with Chao1 and Shannon indices. (**B**) Beta-diversity of bacterial transcripts using the Bray-Curtis dissimilarity index and PERMANOVA statistical method. Each graph is a comparison of the -pre and -post diversity at the family level within each treatment group. From left to right: EscC, Eae, EscC+Eae, and adjuvant-only. (**C**) Relative abundances at the family level from -pre and -post treatment groups. *-pre*, baseline samples before immunization; *-post*, post-immunization sample.

Although EHEC is pathogenic to humans, there are commensal strains of *E. coli* that reside as normal members of the microbiota. To guaranty that vaccines formulated against EHEC do not affect these commensals, antigens that are not shared between pathogenic and commensal strains are preferable. Both proteins used for this vaccine study – EscC and Eae – are encoded by the LEE [26], which is only known to be present in pathogenic strains; however, we confirmed that the AuNP-proteins and the adjuvants did not alter the mouse microbiome composition. For this study, C57BL/6 mice were subcutaneously immunized with AuNPs conjugated to either EscC, Eae, or a combination of the antigens, along with the adjuvants. Mice immunized with unconjugated AuNPs mixed with adjuvants were used as controls. The full regimen used, and the antigen selection, were described in our previous publication [19]. Feces were collected before immunization and 2 weeks following the last dose. Fecal DNA was used for 16S rRNA gene sequencing and analysis. Chao1 and Shannon indices revealed there were no significant changes in alpha diversity within any of the vaccine groups before (pre) and after vaccination (post), however there was a downward trend in the Shannon diversity index in AuNP-EscC immunized mice (Fig. 1A). The beta diversity within each group pre- and post-vaccination did not vary significantly, except in the AuNP-EscC vaccinated animals (p = 0.048) (Fig. 1B).

Taxonomic analysis of each vaccine group showed some differences in the relative abundances of various bacterial families, including in the adjuvant-only group (Fig. 1C). A list of the families that were significantly differentially abundant pre- and post-immunization are listed in Table 1, with the associated p-value and indicating if the family was increased (I) or decreased (D) following immunization. *Lactobacillaceae* was the most abundant family in all animal groups, with a noticeable, but not statistically significant, increase trend seen after AuNP-EscC immunization (pre: 53.4%; post: 71.5%) (Fig. 1C). This change was associated with a significant decrease in *Lachnospiraceae* species (Fig. 1C, Table 1). Furthermore, the relative abundance of *Clostridiaceae* was significantly reduced following immunization with each antigen alone [EscC (pre: 3.4%; post: 0.3%); Eae (pre: 1.7%; post: 0.1%)] and EscC + Eae together (pre: 2.4%; post: 0.5%), with this being the only family differentially abundant in the combo group (Fig. 1C, Table 1). Animals receiving the adjuvant-only mostly had an increase in abundance of certain families, except for *Peptostreptococaceae*, which decreased (pre: 5.49%; post: 2.7%) (Fig. 1C, Table 1).

### AuNP-EscC immunized mice had no significant cage-to-cage differences in microbiota composition.

**Figure 2:**
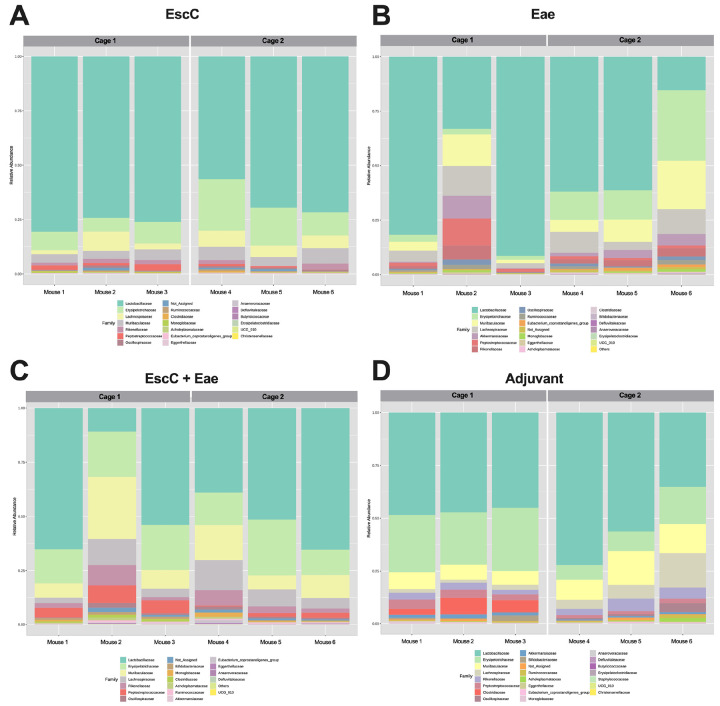
Fecal microbiome compositions of individual mice treated with AuNP vaccine formulations. Relative abundances at the family level in (**A**) AuNP-EscC, (**B**) AuNP-Eae, (**C**) EscC+Eae, and (**D**) adjuvant-only vaccinated animals. Each chart displays a comparison between Cage 1 and Cage 2 (each housing 3 mice) within each treatment group.

During our vaccine study, AuNP-protein and adjuvant-only treated animals were subsequently infected with 10^6^ CFU of *C. rodentium*. The infection resulted in cage-to-cage variations in pathogen shedding within the same group, i.e., all mice in one cage were highly colonized while mice in the other cage shed much lower concentrations, as we have previously shown [19]. This was not completely surprising, considering it is well-known that C57BL/6 mice display inconsistent colonization by *C. rodentium* [27]. However, due to this observed phenomenon, we sought to examine the potential differences in the microbiome before infection in immunized animals within the same group but housed in different cages. It is important to note that AuNP-EscC immunized mice consistently shed *C. rodentium* at lower concentrations than the adjuvant-only controls and had significantly less organ burden at the end of the study [19]. Both AuNP-Eae immunized mice cages shed similar levels while mice in Cage 1 in both the AuNP-EscC + Eae immunized and adjuvant-only treated groups consistently shed much lower concentrations of *C. rodentium* than Cage 2 [19]. Interestingly, following vaccination with AuNP-EscC, only the relative abundance of *Defluviitaleaceae* was significantly different between Cage 1 and Cage 2 (Fig. 2A), whereas there were multiple differences between the two cages in the other groups, such as *Clostridiaceae* or *Peptostreptococcaceae* (Fig. 2B-D). The average relative abundances for the significantly differentially abundant families for each group and the corresponding p-values are given in Table 2. Overall, this data indicates that mice within the same treatment group can have differences in their microbiota composition, which appeared to be, in our conditions, cage dependent. We then evaluated changes in the fecal microbiome following infection.

### AuNP-EscC immunization protected mice from a reduction in diversity and colonization by C. rodentium.

**Figure 3:**
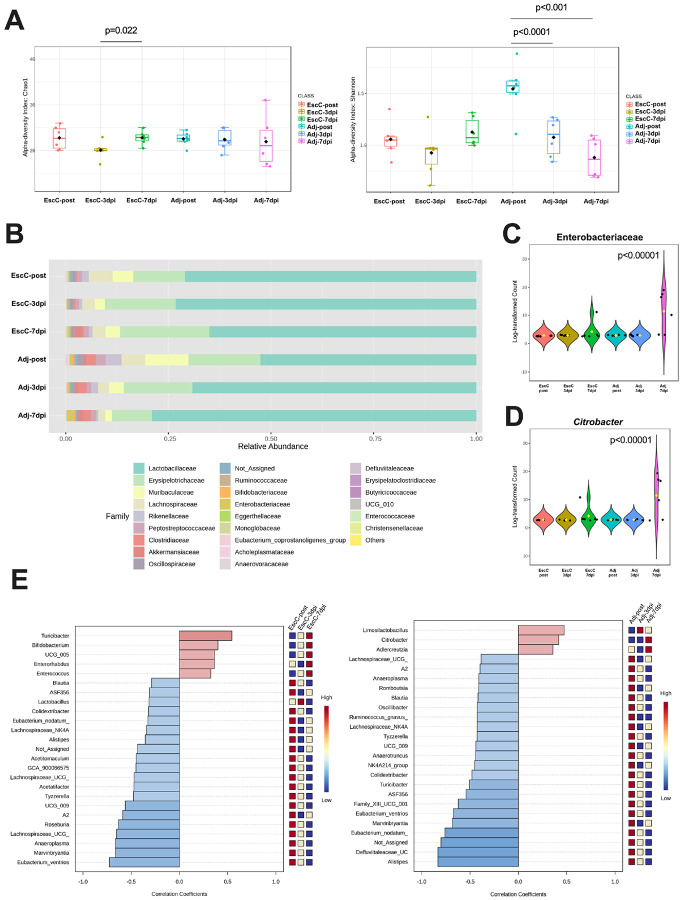
Fecal microbiome composition in immunized or control mice before and following challenge with *C. rodentium*. (**A**) Alpha-diversity of bacterial transcripts at the family level from -post, −3dpi, and −7dpi of AuNP-EscC and adjuvant-only vaccinated groups with Chao1 and Shannon index. (**B**) Relative abundances of bacterial families from -post, −3dpi, and −7dpi of AuNP-EscC and adjuvant-only groups. (**C**) The relative abundance of the *Enterobacteriaceae*family in each treatment group before and after infection. Significance of p<0.00001 measured between adj-7dpi and all other groups. (**D**) The relative abundance of the *Citrobacter* genus in each treatment group before and after infection. Significance of p<0.00001 measured between adj-7dpi and all other groups. (**E**) The top 25 bacterial genera exhibiting the most significant differences throughout the infection timeline, in AuNP-EscC vaccinated (left) and adjuvant-only treated animals (right). *-post*, post-immunization fecal sample; *−3dpi*, fecal samples from 3 days after infection; *−7dpi*, fecal samples from 7 days after infection.

As described above, mice immunized with AuNP-EscC were less colonized by *C. rodentium* and had significantly less organ burden at 14 days post-infection (dpi) compared to the adjuvant-only treated animals. Because *C. rodentium* causes intestinal dysbiosis to aid in its own colonization [10], we wanted to determine if the vaccine could prevent this intestinal microbiota imbalance. The fecal microbiome was analyzed in AuNP-EscC immunized and adjuvant-only treated animals before infection (post-treatment) and at 3dpi and 7 dpi with 10^6^ CFU *C. rodentium*. Although both Chao1 and Shannon indices showed changes in the alpha diversity in AuNP-EscC immunized animals following infection, this was only significant between 3dpi and 7dpi (Chao 1 [p = 0.022] and Shannon index [p = 0.09]) (Fig. 3A). These changes were not significant when comparing EscC-post to either dpi. There was a significant reduction in alpha diversity throughout the infection in adjuvant-only treated animals according to the Shannon index (Adj-post *versus* Adj-3dpi p < 0.0001; Adj-post *versus* Adj-7dpi p < 0.001) (Fig. 3A). Furthermore, both “-post” groups and “−7dpi” differed significantly in the Shannon index as well (EscC-post *versus* Adj-post p = 0.0036; EscC-7dpi *versus* Adj-7dpi p = 0.0317), and this was reflected in the taxonomic analyses. The AuNP-EscC vaccinated animals had few changes in the relative abundances of identified families, with only a noticeable increase in *Akkermansiaceae* after the infection (from 0.008% in EscC-post, to 0.078% in EscC-3dpi, and up to 1.9% in EscC-7dpi), in *Bifidobacteriaceae* (from 0.007% in EscC-post, to 0.11% in EscC-3dpi, and 0.15% in EscC-7dpi), and in *Erysipelotrichaceae* (from 12.62% in EscC-post, to 17.25% in EscC-3 dpi, and to 21.76% in EscC-7dpi). Adjuvant-only treated animals had a bloom of *Lactobacillaceae* (52.58% in Adj-post, 69.15% in Adj-3dpi, and 79% in Adj-7dpi) and concurrent reductions of other families such as *Erysipelotrichaceae* (17.53% in Adj-post, 16.68% in Adj-3dpi, and 9.69% in Adj-7dpi), *Muribaculaceae* (10.5% in Adj-post, 3.64% in Adj-3dpi, and 1.65% in Adj-7dpi), and *Lachnospiraceae* (5.75% in Adj-post, 2.65% in Adj-3dpi, and 1.70% in Adj-7dpi) throughout the infectious process (Fig. 3B). *Lachnospiraceae* was also reduced in the vaccinated group after the infection (5.8% in EscC-post, 3.09% in EscC-3dpi, and 3.14% in Adj-7dpi). There was an increase in *Enterobacteriaceae* in adjuvant-only treated animals (Fig. 3C), as well as a measured increase in *C. rodentium* (Fig. 3D), with statistical significance between Adj-pre and Adj-7dpi in both cases. We further explored these differences using the pattern search analysis in the correlation network of the top 25 genera associated with each group before and after infection. Following infection, both AuNP-EscC immunized and adjuvant-only treated animals were associated with increases and decreases of certain genera (Fig. 3E). For example, *Turicibacter* increased with time in the vaccinated group, while it decreased over the infection course in the control animals. Others like *Alistipes, Blautia*, and members of the *Lachnospiraceae* family exhibited a reduction with time in both treatment groups (Fig. 3E). Most importantly, however, it is the increase in *Citrobacter* That was only associated with adjuvant-only treated animals, confirming the reduction in colonization by the pathogen in the immunized mice (Fig. 3D-E).

## DISCUSSION

Pathogenic *E. coli* remains an important cause of human food-borne diarrheal disease, with some strains able to cause more severe sequalae like HUS or even death in susceptible populations (reviewed in [28]). In cases in which antimicrobial treatment is available because of a sensitivity profile of the bacterium, it is contraindicated when EHEC are the source of the infection since antibiotics are known to increase the production of the Stx [29-31]. Lack of a prophylactic vaccine for human use to protect against EHEC is associated with a myriad of challenges, including the lack of reliable animal for testing [32] and the antigen similarity of the pathogenic strains with the commensal ones, limiting antigenic options. There is also a gap in knowledge regarding how these vaccines impact the gut microbiota, despite its known role in modulating EHEC pathogenesis. A further limitation is age-related fluctuations in the microbiome, which has been proven to affect susceptibility of the host to enteric infections, and how this could affect long-term vaccine studies [33].

Previously, our lab designed a vaccination protocol to test the efficacy of a AuNPs coupled with different EHEC antigenic proteins that are not present in commensal *E. coli*. Although we demonstrated different levels of protection with these formulations after animal immunization and challenges [16-19], all the combinations and routes tested elicited a strong humoral response. Therefore, we questioned if the individual and collective microbiomes of the different groups in our most recent vaccine trial had a significant impact on infection clearance.

The alpha-diversity of the animal groups both before and after vaccination showed no significant differences, indicating that the richness (Chao1) and the evenness (Shannon) of the groups are comparable (Fig. 1A) This is similar to what has been demonstrated by other researchers [14]. However, the beta-diversity, as a measurement of the similarity between groups, showed significant differences only in the AuNP-EscC vaccinated group (Fig. 1B). This indicates there are no differences attributed to the injection route or adjuvants used but only to the antigen, making the chosen vaccination regimen appropriate in those terms. Additionally, all vaccinated animals exhibited a significant reduction in the abundance of *Clostridiaceae* after the vaccination, which was not observed in the control (adjuvant only) group (Table 1). A higher presence of *Clostridium* spp. is commonly associated with adverse effects on normal gastrointestinal (GI) activity [34] and linked with proinflammatory cytokines [35]. However, it has been shown that its prevalence differs from tissue and feces [35].

During our vaccine studies, we found different protection among animals from the same group [19]. We also tested our vaccines against a higher dose of *C. rodentium* (10^9^ CFU), but did not observe the same variability in colonization, with animals in the same group shedding comparable concentrations of bacteria, regardless of cage. We hypothesize that in the high infectious dose, the number of bacteria that colonized the GI tract was too elevated to be outcompeted by the commensal microorganisms, and because of that, the differences are more obvious in the low infectious dose. This was our rationale in only analyzing the microbiome composition in mice subsequently infected with the lower dose. Furthermore, the intestinal microbiome has been extensively proven to be variable among individuals, and factors such as age, diet, and other environmental influences, including geographical location, are known to effect the composition [36]. Therefore, we compared the individual mouse’s fecal microbiome within each group prior to infection to help determine if this could explain the variability in colonization, considering all vaccine formulations elicited similar antibody responses in all immunized animals. The animals vaccinated with EscC and Eae antigens alone exhibited similar shedding and organ burden among animals in the same group [19] and were the groups that exhibited fewer microbiome differences between both cages (Fig. 2A-B, Table 2). In control groups and AuNP-EscC-Eae vaccinated animals, Cages 1 exhibited lower bacterial burden than Cages 2, and in both cases, they shared a higher abundance of families *Clostridiaceae* and *Peptostreptococcaceae* (Fig. 2C-D, Table 2). Some species of *Clostridium* have been reported as potential probiotics that can control the gut inflammatory response, so they might be able to have preventive effects on EHEC infections [28]. Also, *Peptostreptococcaceae* is usually more abundant in healthy individuals' gut and helps in maintaining homeostasis [37]. Overall, our data suggests that variations in the gut microbiota from one individual to the next could have implications in vaccine effectiveness and should be explored further when testing novel vaccines for enteric pathogens.

As previously mentioned, the AuNP-EscC immunized mice showed the lowest burden following infection [19] and was also the only group that exhibited more differences in the microbiome after vaccination (Fig. 1B). These differences, however, were more homogeneous among the animals (Fig. 2A). Because of the significant reduction in colonization compared to the control group that was not seen in the other vaccine groups, we only compared the microbiome between those groups before (-post immunization) and after (−3dpi, −7dpi) infection. There was a clear and significant difference in bacterial richness in the fecal microbiota after infection in both groups, but more noteworthy in the control than the vaccinated group (Fig. 3A). There was also a difference in the evenness, probably due to the high concentrations of *Citrobacter* (Fig. 3D). Specifically, differences in the abundance of families were seen between the AuNP-EscC vaccinated and the control animals (Fig. 3B). AuNP-EscC immunized animals exhibited an increase of *Akkermansiaceae* and *Bifidobacteriaceae* relative abundances after the infection. However, the adjuvant group showed only an increase in *Lactobacillaceae* but a reduction in *Maribaculaceae* and *Lachnospiraceae*. Some *in vivo* studies have shown a protective effect of probiotics in EHEC-challenged animals, which they linked with higher levels of *Akkermansiaceae* and *Lachnospiraceae* in the treated animals [38]. In our study, when we analyzed the trends at the genus level, there was a clear increase of *Bifidobacterium* in AuNP-EscC vaccinated animals after the infection, which has been reported to inhibit the growth of EHEC and reduce the production of Stx in animal models [28]. Nevertheless, the microbiome is a complex community, and our evidence suggests that it should be considered during vaccination due to its proven roles in protection against enteric infection.

## CONCLUSIONS

With this work, we have evaluated the gut microbiota modifications in animals that were vaccinated with AuNPs conjugated to EHEC antigens and then subsequently challenged with the mouse pathogen *C. rodentium*. Although there are limitations to this work, in terms of the number of animals used and that only the fecal microbiota was analyzed, it can pave the way for future studies and does highlight the importance of commensal microbiota in resisting colonization of bacterial pathogens. However, as our lab and others have already demonstrated, robust antibody production is not always a direct indication of protection, at least, against EHEC. Future work is focused on optimizing our vaccines to maximize their efficacy against enteric pathogens in mice and understanding how microbiome maintenance can apply to humans in protection against EHEC.

## Figures and Tables

**Table 1:** Significant differentially abundant families pre- and post-immunization.

Group	Family	p-value	I or D From Pre- Vaccination
EscC	*Bifidobacteriaceae*	0.015152	D
	*Clostridiaceae*	0.004329	D
	*Eubacterium_coprostanoligenes_group*	0.025974	I
	*Lachnospiraceae*	0.025974	D
	*Oscillospiraceae*	0.008658	D
Eae	*Butyricicoccaceae*	0.015152	D
	*Clostridiaceae*	0.002165	D
	*Eggerthellaceae*	0.041126	I
	*Erysipelatoclostridiaceae*	0.002165	I
	*Monoglobaceae*	0.004329	I
	*Rikenellaceae*	0.002165	I
EscC + Eae	*Clostridiaceae*	0.008658	D
Adj	*Acholeplasmataceae*	0.025974	I
	*Anaerovoracaceae*	0.015152	I
	*Bifidobacteriaceae*	0.015152	I
	*Defluviitaleaceae*	0.062771	I
	*Eggerthellaceae*	0.008658	I
	*Muribaculaceae*	0.041126	I
	*Peptostreptococcaceae*	0.041126	D

*I*, increased in post-immunization compared with pre-vaccination; D, decreased in post-immunization compared with pre-vaccination.

**Table 2:** Significant differentially abundant families post-immunization.

		Average Relative Abundance (%)		
Group	Family	Cage 1	Cage 2	p-value
EscC	*Defluviitaleaceae*	0.01	0.03	0.03351
Eae	*Clostridiaceae*	0.02	0.16	0.007693
	*Eubacterium_coprostanoligenes_group*	0.15	1.02	0.027702
EscC + Eae	*Bifidobacteriaceae*	1.03	0.30	0.010424
	*Clostridiaceae*	1.00	0.04	0.018588
	*Peptostreptococcaceae*	6.18	1.50	0.013972
Adj	*Akkermansiaceae*	1.43	0.69	0.012242
	*Anaerovoracaceae*	0.05	0.12	0.018279
	*Butyricicoccaceae*	0.00	0.02	0.02549
	*Clostridiaceae*	5.33	0.03	0.023607
	*Erysipelotrichaceae*	27.23	11.31	0.010853
	*Muribaculaceae*	7.22	13.04	0.042468
	*Peptostreptococcaceae*	3.79	1.65	0.023874

Comparison of the relative abundances of families between both cages in which animals in the same group were housed.

## References

[1] Afzaal M, Saeed F, Shah YA, Hussain M, Rabail R, Socol CT, Human gut microbiota in health and disease: Unveiling the relationship. Front Microbiol. 2022;13:999001. 36225386 10.3389/fmicb.2022.999001 PMC9549250

[2] Kaper JB, Nataro JP, Mobley HLT. Pathogenic *Escherichia coli*. Nat Rev Microbiol 2004 2:2. 2004;2:123–40. 10.1038/nrmicro818 15040260

[3] Iacob S, Iacob DG, Luminos LM. Intestinal microbiota as a host defense mechanism to infectious threats. Front Microbiol. 2019;10 JAN:426119. 10.3389/fmicb.2018.03328 PMC6362409 30761120

[4] Khan I, Bai Y, Zha L, Ullah N, Ullah H, Shah SRH, Mechanism of the Gut Microbiota Colonization Resistance and Enteric Pathogen Infection. Front Cell Infect Microbiol. 2021;11:716299. 35004340 10.3389/fcimb.2021.716299 PMC8733563

[5] Takao M, Yen H, Tobe T. LeuO enhances butyrate-induced virulence expression through a positive regulatory loop in enterohaemorrhagic *Escherichia coli*. Mol Microbiol. 2014;93:1302–13. 25069663 10.1111/mmi.12737

[6] Melton-Celsa AR. Shiga Toxin (Stx) Classification, Structure, and Function. Microbiol Spectr. 2014;2. 10.1128/microbiolspec.EHEC-0024-2013 PMC4270005 25530917

[7] Zumbrun SD, Melton-Celsa AR, Smith MA, Gilbreath JJ, Merrell DS, O’Brien AD. Dietary choice affects Shiga toxin-producing *Escherichia coli* (STEC) O157:H7 colonization and disease. Proc Natl Acad Sci U S A. 2013;110:E2126–33. 23690602 10.1073/pnas.1222014110 PMC3677460

[8] Roxas JL, Koutsouris A, Bellmeyer A, Tesfay S, Royan S, Falzari K, Enterohemorrhagic *E. coli* alters murine intestinal epithelial tight junction protein expression and barrier function in a Shiga toxin independent manner. Lab Invest. 2010;90:1152–68. 20479715 10.1038/labinvest.2010.91 PMC2912457

[9] O’Brien AD, Mohawk KL. Mouse models of *Escherichia coli* O157:H7 Infection and Shiga Toxin Injection. J Biomed Biotechnol. 2011;2011. 10.1155/2011/258185 PMC3022220 21274267

[10] Hopkins EGD, Roumeliotis TI, Mullineaux-Sanders C, Choudhary JS, Frankel G. Intestinal epithelial cells and the microbiome undergo swift reprogramming at the inception of colonic *Citrobacter rodentium* infection. mBio. 2019;10:1–19. 10.1128/mBio.00062-19 PMC6445932 30940698

[11] Yadav M, Chauhan NS. Microbiome therapeutics: exploring the present scenario and challenges. Gastroenterol Rep (Oxf). 2022;10. 10.1093/gastro/goab046 PMC8972995 35382166

[12] Lynn DJ, Benson SC, Lynn MA, Pulendran B. Modulation of immune responses to vaccination by the microbiota: implications and potential mechanisms. Nat Rev Immunol. 2021;22:33–46. 34002068 10.1038/s41577-021-00554-7 PMC8127454

[13] Zimmermann P. The immunological interplay between vaccination and the intestinal microbiota. NPJ Vaccines. 2023;8. 10.1038/s41541-023-00627-9 PMC9947885 36823142

[14] Hays MP, Ericsson AC, Yang Y, Hardwidge PR. Vaccinating with conserved *Escherichia coli* antigens does not alter the mouse intestinal microbiome. BMC Res Notes. 2016;9:1–7. 27514618 10.1186/s13104-016-2208-y PMC4981990

[15] Rojas-Lopez M, Monterio R, Pizza M, Desvaux M, Rosini R. Intestinal pathogenic *Escherichia coli*: Insights for vaccine development. Front Microbiol. 2018;9 MAR:333650. 10.3389/fmicb.2018.00440 PMC5869917 29615989

[16] Sanchez-Villamil JI, Tapia D, Torres AG. Optimization of Multivalent Gold Nanoparticle Vaccines Eliciting Humoral and Cellular Immunity in an *In Vivo* Model of Enterohemorrhagic *Escherichia coli* O157:H7 Colonization. mSphere. 2022;7. 10.1128/msphere.00934-21 PMC8769200 35044806

[17] Sanchez-Villamil JI, Tapia D, Torres AG. Development of a gold nanoparticle vaccine against Enterohemorrhagic *Escherichia coli* O157:H7. mBio. 2019;10. 10.1128/mBio.01869-19 PMC6692519 31409688

[18] Bowser S, Melton-Celsa A, Chapartegui-González I, Torres AG. Efficacy of EHEC gold nanoparticle vaccines evaluated with the Shiga toxin-producing *Citrobacter rodentium* mouse model. Microbiol Spectr. 2023;12:e02261. 38047703 10.1128/spectrum.02261-23 PMC10783022

[19] Bowser S, Melton-Celsa A, Chapartegui-González I, Torres AG. Further Evaluation of Enterohemorrhagic *Escherichia coli* Gold Nanoparticle Vaccines Utilizing *Citrobacter rodentium* as the Model Organism. Vaccines (Basel). 2024;12:508. 38793759 10.3390/vaccines12050508 PMC11125983

[20] Gansheroff LJ, Wachtel MR, O’Brien AD. Decreased adherence of enterohemorrhagic *Escherichia coli* to HEp-2 cells in the presence of antibodies that recognize the C-terminal region of intimin. Infect Immun. 1999;67:6409–17. 10569757 10.1128/iai.67.12.6409-6417.1999 PMC97049

[21] John Turkevich B, Cooper Stevenson P, Hillier J. A Study of the Nucleation and Growth Processes in the Synthesis of Colloidal Gold. Discuss Faraday Soc. 1951;11:55–75.

[22] Klindworth A, Pruesse E, Schweer T, Peplies J, Quast C, Horn M, Evaluation of general 16S ribosomal RNA gene PCR primers for classical and next-generation sequencing-based diversity studies. Nucleic Acids Res. 2013;41:e1. 22933715 10.1093/nar/gks808 PMC3592464

[23] Quast C, Pruesse E, Yilmaz P, Gerken J, Schweer T, Yarza P, The SILVA ribosomal RNA gene database project: improved data processing and web-based tools. Nucleic Acids Res. 2013;41:D590–596. 23193283 10.1093/nar/gks1219 PMC3531112

[24] Chong J, Liu P, Zhou G, Xia J. Using MicrobiomeAnalyst for comprehensive statistical, functional, and meta-analysis of microbiome data. Nat Protoc. 2020;15:799–821. 31942082 10.1038/s41596-019-0264-1

[25] Lu Y, Zhou G, Ewald J, Pang Z, Shiri T, Xia J. MicrobiomeAnalyst 2.0: comprehensive statistical, functional and integrative analysis of microbiome data. Nucleic Acids Res. 2023;51:W310–8. 37166960 10.1093/nar/gkad407 PMC10320150

[26] Elliott SJ, Sperandio V, Giron JA, Shin S, Mellies JL, Wainwright L, The locus of enterocyte effacement (LEE)-encoded regulator controls expression of both LEE- and non-LEE-encoded virulence factors in Enteropathogenic and Enterohemorrhagic *Escherichia coli*. Infect Immun. 2000;68:6115–26. 11035714 10.1128/iai.68.11.6115-6126.2000 PMC97688

[27] Mundy R, Macdonald TT, Dougan G, Frankel G, Wiles S. *Citrobacter rodentium* of mice and man. Cell Microbiol. 2005;7:1697–706. 16309456 10.1111/j.1462-5822.2005.00625.x

[28] Lee KS, Jeong YJ, Lee MS. *Escherichia coli* Shiga Toxins and Gut Microbiota Interactions. Toxins (Basel). 2021;13:416. 34208170 10.3390/toxins13060416 PMC8230793

[29] Freedman SB, Xie J, Neufeld MS, Hamilton WL, Hartling L, Tarr PI, Shiga Toxin–Producing *Escherichia coli* Infection, Antibiotics, and Risk of Developing Hemolytic Uremic Syndrome: A Meta-analysis. Clin Infect Dis. 2016;62:1251–8. 26917812 10.1093/cid/ciw099 PMC4845788

[30] Wong CS, Jelacic S, Habeen RL, Watkins SL, Tarr PI. The Risk of Hemolytic-Uremic Syndrome after Antibiotic Treatment of *Escherichia coli* O157:H7 Infections. N Engl J Med. 2000;342:1930–6. 10874060 10.1056/NEJM200006293422601 PMC3659814

[31] Kakoullis L, Papachristodoulou E, Chra P, Panos G. Shiga toxin-induced haemolytic uraemic syndrome and the role of antibiotics: a global overview. J Infect. 2019;79:75–94. 31150744 10.1016/j.jinf.2019.05.018

[32] Ritchie JM. Animal Models of Enterohemorrhagic *Escherichia coli* Infection. Microbiol Spectr. 2014;2. 10.1128/microbiolspec.EHEC-0022-2013 26104195

[33] Walrath T, Dyamenahalli KU, Hulsebus HJ, McCullough RL, Idrovo JP, Boe DM, Age-related changes in intestinal immunity and the microbiome. J Leukoc Biol. 2021;109:1045–61. 33020981 10.1002/JLB.3RI0620-405RR PMC8139861

[34] Singh R, Zogg H, Wei L, Bartlett A, Ghoshal UC, Rajender S, Gut Microbial Dysbiosis in the Pathogenesis of Gastrointestinal Dysmotility and Metabolic Disorders. J Neurogastroenterol Motil. 2021;27:19–34. 33166939 10.5056/jnm20149 PMC7786094

[35] Borgognone A, Elizalde-Torrent A, Casadellà M, Romero L, Escribà T, Parera M, Vaccination with an HIV T-cell immunogen induces alterations in the mouse gut microbiota. NPJ Biofilms Microbiomes. 2022;8:1–9. 36585401 10.1038/s41522-022-00368-y PMC9801356

[36] Hall AB, Tolonen AC, Xavier RJ. Human genetic variation and the gut microbiome in disease. Nat Rev Genet. 2017;18:690–9. 28824167 10.1038/nrg.2017.63

[37] Cho S, Kumar SS, Ramirez S, Valientes R, Kim IH. Dietary eubiotics of microbial muramidase and glycan improve intestinal villi, ileum microbiota composition and production trait of broiler. J Anim Sci Biotechnol. 2024;15:1–14. 38594781 10.1186/s40104-024-01010-x PMC11005127

[38] Bao CL, Liu SZ, Shang ZD, Liu YJ, Wang J, Zhang WX, *Bacillus amyloliquefaciens* TL106 protects mice against enterohaemorrhagic *Escherichia coli* O157:H7-induced intestinal disease through improving immune response, intestinal barrier function and gut microbiota. J Appl Microbiol. 2021;131:470–84. 33289241 10.1111/jam.14952

